# COVID-19 sample management: experiences of Harare City, 2021

**DOI:** 10.11604/pamj.2022.41.339.33514

**Published:** 2022-04-27

**Authors:** Emmaculate Govore, Talent Bvochora, Hilda Bara, Prosper Chonzi

**Affiliations:** 1 Beatrice Road Infectious Diseases Hospital, Harare City, Department of Health, Harare, Zimbabwe

**Keywords:** Sample management, COVID-19, Harare

## Abstract

COVID-19 sample management is pivotal in controlling the pandemic. Results of 28/45 (62.2%) cases identified at a high school COVID-19 outbreak in Harare’s Northern district were not reported within the recommended 24 hours of notification. This leads to delayed patient management. We evaluated the sample management system for COVID-19 in Harare City. A descriptive cross-sectional study was conducted in Harare City. Health care workers involved in COVID-19 sample management at a high school outbreak in the Northern district namely clinicians, laboratory, environmental and administrative personnel were purposively sampled. Interviewer-administered questionnaires were used to collect data. Quantitative data were analyzed using Epi info version 7. Medians and proportions were generated. A 3-point Likert Scale was used to measure knowledge levels of health care workers on COVID-19 sample management. Thirty health care workers were interviewed and females were 20/30 (66%). Participants had not been trained in sample management. Overall knowledge level was good for 7/30 (23%) of the participants. Of the samples collected, 156/451 (34.6%) were wrongly sent to the national microbiology laboratory and 53/156 (34.0%) of the results were received. Sample management of COVID-19 samples in Harare City was found to affect patient management because of poor knowledge of healthcare workers, lack of transportation and communication means. The need for training cadres involved in the management process and availing adequate resources can improve turnaround time of results hence patient management.

## Introduction

Coronavirus disease 2019 (COVID-19) is caused by Severe Acute Respiratory Corona Virus-2 (SARS-CoV-2) [1]. The disease was first reported in December 2019 in Wuhan, China. COVID-19 has spread globally, is now a pandemic and Zimbabwe has not been spared. Globally, as of March 31, 2021, there have been 128,540,982 confirmed cases of COVID-19, including 2,808,308 deaths [2]. Zimbabwe, as of March 23, 2021 had recorded 36,717 confirmed cases, and 1,516 deaths, and Harare had 12,915 confirmed cases and 589 deaths [3].

COVID-19 disease symptoms are non-specific and 40% of the people who are positive for the viral disease are asymptomatic [4]. It is therefore important to use laboratory tests to identify SARS-Cov-2 infections. Sample management indirectly becomes key in identifying and managing the disease. Sample management involves specimen collection, handling, transportation, processing and results feedback.

A study in Uganda found that there was a lack of timeliness in laboratory testing due to challenges which included lack of literacy among the workforce, inadequate digital infrastructure and internet access and lack of coordination between the health systems [5]. The number of existing laboratories, the availability of trained human resources and the reporting structures were noted to be challenges in sample management processes [6]. Recommendations have been made on the importance of timely communication between facility health workers and laboratory professionals to ensure collaboration, through memos, phone and email services [7].

Naso-pharyngeal specimen collection in Harare City is done at testing sites. Samples requiring rapid antigen testing are processed at the site of collection, whilst those requiring polymerase chain reaction testing are sent to the main laboratory at Beatrice Road Infectious Diseases hospital (BRIDHL). Samples that cannot be tested at BRIDHL are referred to the National Reference Laboratory (NMRL). The turnaround time of COVID-19 results is 24 hours. Notwithstanding the outlined procedures above, results of 28/45 (62.2%) of cases identified at a high school COVID-19 outbreak in Harare´s Northern district were not reported within the 24hours of notification. This led to delayed patient management and measures to control the spread of the disease. We, therefore, saw the need to describe the sample management for COVID-19 in the city. This report would help identify gaps and come up with recommendations in sample management for COVID-19 in the city.

## Methods

**Study design and setting:** a descriptive cross-sectional study was conducted in Harare City health department in Harare Metropolitan Province of Zimbabwe. The city is divided into four districts, Northern, Southern, Eastern, and Western districts which are serviced by one functional laboratory.

**Study population:** the study population were clinicians, laboratory, and environmental personnel who were involved in COVID-19 sample management at the high school outbreak in the Northern district.

**Sample size and sampling technique:** all team members of the rapid response for Harare City Northern district that participated in the high school COVID-19 outbreak were purposively sampled.

**Data collection and tools:** data was collected using an interviewer-administered questionnaire to assess sample collection, transportation, and sample processing factors that contributed to the late and non-return of COVID-19 PCR results. Checklists were used to verify the availability of resources.

**Data analysis:** quantitative data were analysed using Epi info version 7. Medians and proportions were generated.

**Knowledge of healthcare workers on COVID-19 sample management:** a 3 point Likert Scale was used to measure knowledge levels. Those who answered correctly 2 or fewer questions were recorded as having poor knowledge, those who answered 3-4 questions were recorded as having fair knowledge, and those who answered correctly 5-7 questions were recorded as having good knowledge on COVID-19 sample management.

## Results

**Demographic and occupational characteristics of the respondents:** a total of 30 health care workers were interviewed and the majority were females 20/30 (66%). Nurses were 7/30 (23%) and emergency medical technicians were 5/30 (17%). The median age in years was 38 (Q1=30, Q3=45) and the median years in service was 8 years (Q1=4, Q3=10) (Table 1).

**Table 1:** demographic characteristics and knowledge levels of respondents

Category	Frequency n=30	Percent
**Sex**		
Female	20	66%
Male	10	34%
**Designation**		
Laboratory	10	34%
Nurses	7	23%
EMTs	5	17%
Environment	4	13%
Doctor	4	13%
**Health care worker knowledge levels on COVID-19 sample management**		
**Variable**	**Frequency n=30**	**Percent**
Knowledge of type of specimen	30	100%
Knowledge of notification period	23	77%
Knowledge on triple packing	22	73%
Knowledge on filling transmittal forms	12	40%
Knowledge on sample handling	10	33%
Knowledge on where samples are sent	7	23%
Knowledge on communication channels	9	30%
Overall knowledge levels		
Good (score= 5-7)	7	23%
Fair (score= 3-4)	11	37%
Poor (score= 1-2)	12	40%

**Health care workers' knowledge levels on COVID-19 sample management:** all participants had been trained in COVID 19 management in general but not specifically on sample management. All the participants knew the type of specimen to collect. Knowledge of triple packaging was 22/30 (73%) but less than half of the participants knew how to fill in the transmittal forms. Overall knowledge level was good for 7/30 (23%) of the participants (Table 1).

**Sample collection factors that contribute to late/non-return of results:** sample collection inputs were available in adequate amounts, which included human resources and testing kits. A total of 451 samples were collected during the high school outbreak. Of these samples, 267/451 (59.2%) were sent to the national virology laboratory, 156/451 (34.6%) were sent to the national microbiology laboratory, 28/451 (6.2%) were sent to BRIDHL and 1/451 (0.2%) sample went to a private laboratory (Table 2).

**Table 2:** factors that contribute to late/non-return of laboratory results

Sample collection factors				
		Target	Available	Gap
Human resources		15	15	0
Transport media		451	1000	0 surpassed
Swab		451	1000	0 surpassed
Forms		451	800	0 surpassed
**Transportation factors**				
Transport services		2	1	1
Triple packing kit		451	20	431
Transmittal forms		60	250	0 surpassed
Communication services		2	0	2
**Sample processing factors**				
Human resources		2	2	0
Reagents		451	800	0 surpassed
Machines		2	1	1
Sundries (paper for printing)		451	200	251
Backup power source		1	1	0

**Transportation factors that contribute to late/non-return of laboratory results:** there were 20 of the targeted 451 triple packaging kits available. Out of the two required vehicles, there was one available for transportation of samples, which was not dedicated to COVID-19 samples. Communication services such as mobile phones, wifi, and data were not available (Table 2).

**Personnel and reagents for processing COVID-19 samples were available:** backup generator and a 16 module Gene TM Xpert were also available. Sundries which included paper for printing and printer toner were 200 and 451 were required (Table 2). Of the 451 samples collected, 189/451 (41.9%) results were received. Of the samples collected, 108/267 (40.4%) results were received from the national virology laboratory, 53/156 (34.0%) from the national microbiology laboratory, 28/28 from BRIDHL, and 1/1 from a private laboratory.

## Discussion

The study we conducted on the evaluation of the COVID-19 sample management found that in Harare City health department there was late or non-return of COVID-19 results. Healthcare workers’ knowledge on sample management was also poor, especially on filling transmittal forms and communication channels. Samples that were supposed to be sent to BRIDHL were wrongly sent to the national reference laboratory. Transportation factors which included availability of transport, triple packaging kits and communication means affected the turnaround time of the results.

In Harare City, the laboratory is centralized and samples are sent there from testing centers. In our study, it was reported that some samples were transported to the wrong laboratory. This could have been because of the poor knowledge on the referral path, filling of transmittal forms and the communication channel. Shortage of triple packaging could have led to late transportation of the samples. With only one vehicle available which is not dedicated to collecting COVID-19 samples, this could have contributed to the late and no transportation of samples. Inability to communicate because of the unavailability of mobile phones and wifi could have also contributed to the late transportation of samples. Similar to our study, other studies found lack of knowledge among healthcare workers, resources shortages and ineffective communication systems contributing to poor management of laboratory samples [5,8]. According to WHO, health workers who package and drivers involved in the transportation of samples should be trained in the safety and good maintenance of samples [9].

**Limitations:** this study was done over a certain period, after an outbreak at a high school. There could be different conditions after some time.

## Conclusion

Sample management of COVID-19 samples in Harare City was found to affect patient management because of poor knowledge of the health care workers, and lack of transportation and communication means. The need for training of cadres involved in this management process, availing of adequate resources can improve the turnaround time of results hence patient management.

### What is known about this topic

- The sample management process follows a specific pathway that can be adopted and adapted.

### What this study adds

- This study adds supportive evidence that the late or non-return of results is affected by transportation factors and by poor knowledge of health care workers on sample management processes.
